# Pilot fatigue survey: A study of the mutual influence among fatigue factors in the “work” dimension

**DOI:** 10.3389/fpubh.2023.1014503

**Published:** 2023-02-02

**Authors:** Sun Jun-Ya, Sun Rui-Shan

**Affiliations:** College of Safety Science and Engineering, Civil Aviation University of China, Tianjin, China

**Keywords:** pilot fatigue, “work” dimension, fatigue factors, mutual influence, long-haul flights

## Abstract

**Background:**

Fatigue risk management for pilots has received increasing attention. The existing fatigue management systems have detailed descriptions of the factors and the mutual influences among the factors that affect the dimension of “sleep”, which is one of the most important causes of fatigue. However, the analysis of the influencing factors of the “work” dimension of fatigue causes has not been very detailed or accurate, especially the exploration of the mutual influence among many fatigue-influencing factors in the “work” dimension.

**Objective:**

The purpose of this study was to explore the mutual influence among fatigue-influencing factors related to the “work” dimension in the analysis of pilot fatigue causes.

**Methods:**

This study designed a questionnaire on the dimension of “work” in the causes of pilot fatigue and collected a total of 270 feedback data points from international flight pilots. Based on the questionnaires and data, descriptive statistical analysis, exploratory factor analysis and confirmatory factor analysis were performed to explore the influencing factors and their mutual influences on the “work” dimension of pilot fatigue.

**Results:**

There is a strong, mutual influence relationship among the fatigue causes of long-haul flight pilots – working status, working conditions and working schedules – in the dimension of “work”. The workload only has a strong correlation with the working schedule, and the interaction relationships with the working status or working conditions are weak.

**Conclusion:**

This study analyses the mutual influence among the influencing factors of the “work” dimension of pilot fatigue, and we expect to provide empirical data for pilot fatigue risk management and to help improve fatigue risk management systems.

## 1. Introduction

Fatigue can lead to decreases in pilots' alertness, cognitive ability, judgement, decision-making ability, memory and attention, causing memory omissions, operating errors, decision-making errors, mistakes, and other safety hazards, or it can lead to unconscious drowsiness (1), resulting in the occurrence of “sleeping-in-working” events and other unsafe events, further causing pilots' anxiety, tension, irritability and other adverse psychological stress reactions and negative emotions. In addition, fatigue affects the communication, cooperation and cooperation among crew members, and in severe cases, accidents can occur (2). For example, the Guantanamo Bay accident in 1993 was the first accident in history in which pilot fatigue was considered the main cause. It took a long time for the National Transportation Safety Board (NTSB) investigators to list fatigue as the main cause of this accident because pilot fatigue had rarely been listed as a cause or factor before 1993 (3). Subsequently, fatigue was considered by the accident investigation team to be one of the causes of the accident (4), and it has also become a main concern of NTSB accident investigations (5). In the classified incident reports of the NASA Aviation Safety Reporting System, 52,000 incidents have been clearly classified as being caused by fatigue, accounting for 21% of all incidents (6). Additionally, in a statement by 28 eminent sleep scientists, fatigue was described as the largest identifiable and preventable cause of accidents in transportation operations (fatigue accidents account for 15% to 20% of all accidents) (7). Therefore, alleviating the problem of pilot fatigue is considered to be one of the key determinants for managing and improving flight safety (8).

Fatigue surveys have shown that pilot fatigue is widespread, with a 2011 survey by the British Civil Aviation Pilots Association and the University of London showing that 45% of pilots felt they were “severely fatigued” at work. Forty-three percent of pilots with work fatigue dozed off while flying, and two pilots even fell asleep at the same time while in the air (9). Another United Kingdom (UK) pilot fatigue survey found that 56% of 500 commercial pilots admitted to falling asleep in the cockpit of a plane, with nearly 1/3 saying they woke up to find the copilot also asleep (10).

Although the problem of pilot fatigue is common, there are many problems in the investigation and research of fatigue. For example, there is no unified definition of the concept of fatigue thus far. In terms of industry, the ICAO Fatigue Risk Management System (FRMS) (DOC.9966) defines fatigue as a physiological condition in which the ability to perform mental or physical activities is reduced due to insufficient sleep, prolonged wakefulness, circadian phase and/or heavy workload (mental and/or physical activity). This physiological state can impair people's alertness and ability to perform safety-related operational duties (11). According to the Australian and New Zealand National Road Transport Council (12), the conceptual definition of fatigue in transport usually refers to long periods of wakefulness, long periods of insufficient or inadequate sleep quality, sustained mental or physical effort, disrupted circadian rhythms, insufficient rest periods and environmental stress (such as heat, noise and vibration). In terms of experts and scholars, CRATCOH (13) considers fatigue a marker of a marked decline in the ability to perform tasks and a state of reduced performance; in this state, even in the presence of considerable stimuli, it is uncertain whether a person can be awakened in an emergency. Samuel Strauss (14) defined “fatigue” as a non-pathological state in which maintenance function or workload decreases due to mental or physical stress; this term has been used to describe a range of experiences from sleepiness and tiredness to exhaustion. The Laboratory of Applied Anthropology at the University of Paris V, France, noted that fatigue could be defined as “a series of performances produced by stress and long hours of work beyond a certain limit” (15). In addition, regarding fatigue, many definitions have been proposed by several scholars based on their knowledge in their respective research fields (16–18). Therefore, it remains difficult to determine an accurate and complete definition of “fatigue” thus far because there has been no unified understanding of the mechanism of fatigue.

In addition, in the aviation workplace, there are many factors that lead to fatigue, and the two dimensions sleep and circadian rhythms are generally considered to be the main factors leading to fatigue (19); however, there have been many studies of the causes of fatigue in these two dimensions, and the studies have been relatively complete. Nevertheless, work factors, such as extended working hours and misplaced working schedules, can also lead to severe subjective and physical fatigue, cognitive decline and errors, and safety risks (20); therefore, research on the fatigue-influencing factors of the “work” dimension, such as working status, working conditions, workload and working schedules, is also very important for pilot fatigue risk management. There have also been many in-depth studies of individual factors of the “work” dimension, such as the impact of workload on fatigue (21). However, there have been no targeted reports on the mutual influence among the influencing factors of the “work” dimension, and accurate quantification of the mutual influence among the fatigue-influencing factors of the “work” dimension is equally important for the safety of pilots.

The civil aviation industry has begun to pay more attention to the issue of fatigue. In June 2008, FAA Director Robert A. Sturgell proposed strengthening the management of fatigue at the “New Approach to Fatigue Management” safety forum. U.S. Transportation Secretary Ray LaHood and FAA Administrator Randy Babbitt included pilot fatigue in a call to action for aviation safety following the February 2009 crash of Colgan Air Flight 3407. Questions were listed as a top priority, using the latest fatigue research to create new pilot flight, duty and rest recommendations based on fatigue science. The NTSB also issued a letter of advice to the FAA recommending that the FAA develop guidelines based on experience and scientific evidence for operators to establish fatigue management systems to address human fatigue in aviation operations (22, 23). ICAO added the concept of a FRMS into Annex 6 of international civil aviation standards and recommendations and successively published the “FRMS Operator Implementation Guide” and Doc 9966 “FRMS Supervision Manual”, suggesting that member states implement a FRMS based on scientific principles (11). The FAA of the United States (US) issued Advisory Circular 120-103A on FRMSs (24), and Canada, New Zealand and Australia, which started earlier, have continued to operate a domestic airlines' FRMS and publish explanatory documents on FRMSs. In addition, many countries and regions, such as the European Union and the UK, have also introduced requirements for airlines to operate a FRMS through normative documents or other forms (25). In May 2021, based on the requirements of Part 121 on fatigue risk management, the Civil Aviation Administration (CAA) of China issued an advisory circular, “CCAR Part 121 Fatigue Management Requirements for Certificate Holders” (26), for FRMSs for air operators in accordance with Part 121.

Fatigue risk management for pilots is critical to preventing aircraft accidents, yet most of the text that discusses fatigue factors in the ICAO Oversight Manual on Fatigue Management Methods (Second Edition) (11) is related to sleep (27). There is less of a summary of fatigue causes for other dimensions, which could be related to the continuing research on other dimensions of fatigue. Therefore, this study focuses on the influencing factors of the fatigue “work” dimension and explores the mutual influence among the fatigue-influencing factors in the “work” dimension. Through a targeted and detailed investigation and analysis of the influencing factors of the “work” dimension in the causes of fatigue, we expect to provide suggestions for the civil aviation industry to release more comprehensive fatigue management documents.

## 2. Assumptions about pilot fatigue-influencing factors in the “work” dimension

Pilots often face work characteristics such as long shifts, early shifts, late arrivals, and non-standard working hours, and in many respects, pilots face fatigue factors similar to those encountered by industrial shift workers (28). However, pilots also face many additional factors that are related to the particularities of the civil aviation industry, especially in terms of the “work” dimension. For example, in this study, the influencing factors of the “work” dimension in the cause of pilot fatigue are divided into working status factors, such as complex weather disturbances; working condition factors, such as a narrow cockpit space; workload factors, such as physical and mental loads; and working schedule factors, such as ultralong flight duty.

Regarding the pilot's working status factors, this study does not include personal physiological factors, such as sleep, circadian rhythm, and physical condition, specifically referring to the pilot's working status after being affected by flying, including the impacts of air flow and other meteorological environmental disturbances (29), sudden technical failures (30), support at work (31), flight schedule adjustments, and communication with others (32).

The pilot's working conditions in this study are different from the environmental factors that affect sleep. The focus here is on the working environment, adverse weather conditions, noise, temperature, vibrations, the presence of toxic and harmful substances, improper lighting and other aspects of the working environment; when something does not meet the physical and psychological needs of the staff, it will increase the feeling of fatigue (33–35). In addition, unreasonable environmental factors, such as the unreasonable design of equipment, tools and man-machine interfaces, will cause the working posture of the human body to not perfectly match with the workstation, and work performance will be unreasonable and unsatisfactory, in turn causing physical fatigue, such as greater physical exertion, as well as psychological and mental fatigue caused by work responsibility pressure (36, 37). There are also some social conditions at work, such as business operation pressure, meal quality, and transit rest conditions.

Regarding the pilot's workload factors, this study uses the factors considered on the NASA-TLX (Task Load Index) workload scale (38), and included in the ICAO definition of fatigue is the description of workload as “mental or physical activity”, which is considered a significant cause of fatigue (11). In addition, the complexity of the work and the required personnel proficiency, comprehensive ability, and sophistication will also increase the workload (39). In addition to the time limit to complete the work procedure in the scheduled time, the stress of the pilot during the workload causes psychological changes. To overcome these adverse psychological factors, the human body must make more efforts than before to cope with the work, causing fatigue to be more easily induced.

Regarding the pilot's working schedule factors, Goode (4) found that the probability of a commercial aviation accident increased significantly with increasing duty hours, with 20% of US commercial aviation accidents appearing to occur on duty of 10 h or more. Additionally, staying awake and working for 18.5–21 h can produce performance changes similar to those seen with a blood alcohol concentration of 0.05–0.08% (40). Therefore, the working schedule factors of this study include long-term work. In addition, they also include schedules for night flights (41), schedules with different lengths of transit time (42), and international exemption/non-exemption schedules implemented by the CAA of China during the COVID-19 outbreak (43).

According to the literature review and theoretical analysis, factors of the “work” dimension are also important influencers of pilot fatigue. This view has been agreed upon by many researchers, and there have also been many relevant research conclusions. However, there have been no relevant reports on the mutual influence among the fatigue-influencing factors of the “work” dimension. Therefore, it is necessary to investigate the mutual influence among the factors affecting fatigue in the “work” dimension; however, before further investigation, this study proposes the following hypotheses.

Hypothesis a. Pilot working status and working conditions have a strong mutual influence;
Hypothesis b. Pilot working status and working schedule have a strong mutual influence;
Hypothesis c. Pilot working status and workload have a strong mutual influence;
Hypothesis d. Pilot working conditions and working schedules have a strong mutual influence;
Hypothesis e. Pilot working conditions and workload have strong mutual influence; and
Hypothesis f. Pilot working schedules and workloads have a strong mutual influence.

## 3. Methods

### 3.1. Survey questionnaire design

The contents of the questionnaire used in this study are as follows:

1) Basic personal information about the pilot, mainly including age, pilot level, marital status and number of children, working years and total flying hours, commute time, route type and route area.
2) Working status, mainly including 7 items: meteorological environment and passenger interference risk, technology and failure risk, flight adjustment, job security, and personnel communication.
3) Working conditions, mainly including 13 items, such as noise and other environmental factors, work rhythm disorders, and flight task situation.
4) Workload, mainly including 6 items, including mental, physical and psychological aspects.
5) Working schedule, mainly including 6 items, such as whether an international flight is overnight, a short flight interval and the duty time.

Please refer to the Appendix for the specific questionnaire.

### 3.2. Sample selection and data collection methods

Considering some international airlines in China as an example, on the basis of investigating the actual situation of airline fatigue, fatigue management, fatigue mitigation measures, etc., general statistical analysis and structural equation modeling methods were used and focused on the causes of fatigue of international flight pilots – the fatigue-influencing factors of the “work” dimension. The survey methods of this study were an online survey (using the Questionnaire Star network collection platform) and a mail survey. Through the construction of the FRMS with the airline, the assistance and cooperation of the airline pilots were obtained, and sample questionnaire data were collected.

The questionnaire for this study was employed for data collection at a Chinese airline from October to December 2021, and the pilots of the company's international flights were selected to participate in the scale data collection, excluding the following pilots: (i) any pilot taking melatonin and sleeping pills (due to their affect on circadian rhythms), according to the CAA (2011), the crew cannot take sleeping pills at least 12 hours prior to duty and must be free of any adverse effects prior to duty; (ii) any pilot with an underlying health condition that affects sleep (e.g., chronic fatigue syndrome, depression, seasonal mood disorder, anorexia nervosa); and (iii) any pilot with other current sleep disorders. All participants signed an informed consent form and confidentiality agreement prior to inclusion in the study.

In this study, 100 questionnaires were mailed in a paper version, 89 questionnaires were recovered, 6 invalid questionnaires were excluded, and the effective recovery rate was 83%. The link for 200 questionnaires was sent to the Star Network platform, and a total of 184 questionnaires were collected. Three invalid questionnaires were excluded, and the effective recovery rate was 90.5%. The overall effective recovery rate was 88%.

## 4. Results and discussion

### 4.1. Descriptive statistical analysis

Descriptive statistics mainly included grouping variables and interval division, frequency and percentage statistical analyses. The descriptive statistics of this study included a statistical description of the basic information of long-haul international flight pilots and a statistical description of the factors affecting fatigue in the “work” dimension (including working status, working conditions, workload, and working schedules).

First, through descriptive statistical analysis of the personal basic information part of the pilot's questionnaire results, the summary results, as shown in Table 1 below, demonstrated that copilots accounted for 54.07% of the pilot's job category, and the captains and instructors accounted for the remaining 25.56 and 20.37%, respectively. Therefore, the survey results regarding the pilot's fatigue factors in the “work” dimension in this study focused on the interpretation of the copilot population. In terms of marital status, the proportion that was married was larger, so the survey results focused on the explanation of the fatigue of married pilots. In terms of the number of children, the survey results focused on the pilot groups explained below. In terms of the total flight time, the survey results showed similar explanations for the fatigue of pilot groups with a boundary of 3,500 h. Regarding the route types, the analysis focused on the explanations of the fatigue of pilots flying exempted international routes (exemption/same-night international routes are temporary deviations from certain international routes devised by the CAA of China in 2020 in response to COVID-19 epidemic prevention and control). The analysis also investigated the fatigue status of pilots during the epidemic period and provided basic data for pilot fatigue management during the epidemic period. In terms of the route area, the findings focused on explaining the fatigue of pilots flying to the Americas.

**Table 1:** Statistical analysis of pilots' questionnaires.

**Factors**	**Type**	**Frequency**	**Percentage**
Rank of pilot	Copilot	146	54.07%
	Captain Pilot	69	25.56%
	Instructor	55	20.37%
Marital status	Single	103	38.15%
	Married	158	58.52%
	Divorced	9	3.33%
Number of children	None	116	42.96%
	One	110	40.74%
	Two or more	44	16.30%
Total flight time	< 3500 h	134	49.63%
	≥3500 h	136	50.37%
Line type	Exemption/no overnight	215	79.63%
	Not exempted/overnight	55	20.37%
Route area	Americas	135	50.00%
	Europe	88	32.59%
	Australia	47	17.40%

**Figure 1:**
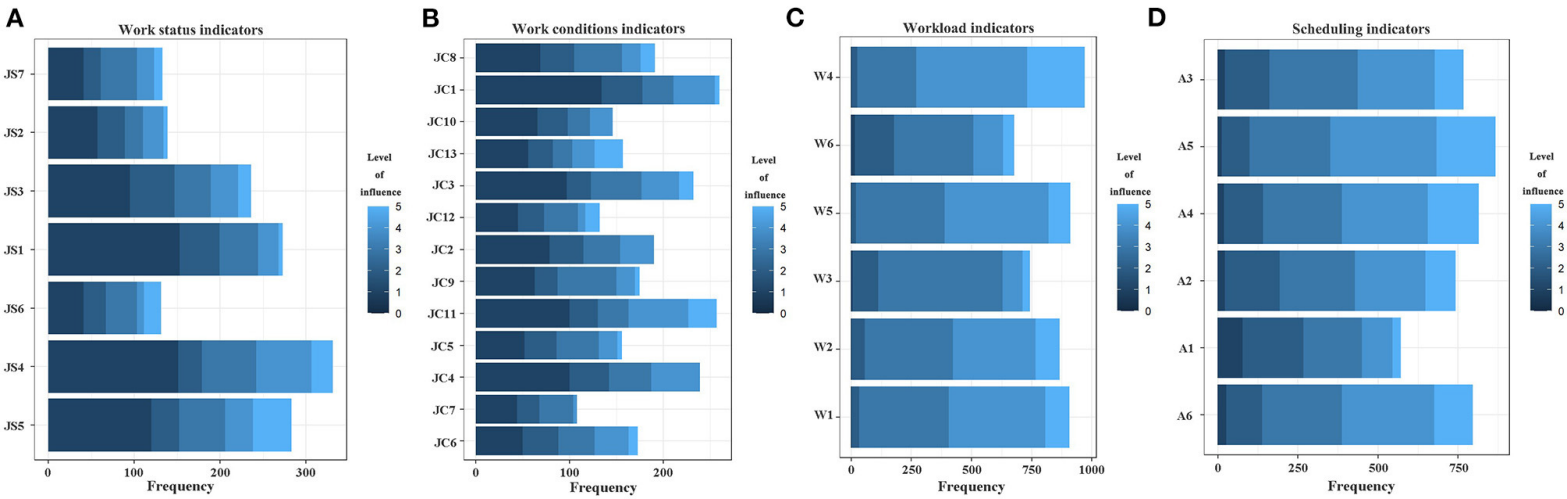
**(A–D)** Statistical analysis of factors affecting fatigue in long-haul flight pilots' “work” dimension.

Figure 1 presents a descriptive statistical analysis of the factors affecting pilot fatigue in terms of the “work” dimension. Figure 1A shows that the “flight plan (temporary) adjustment JS4” impact indicator in the working status had the most feedback about the impact of fatigue, and the impact was also high; it was second only to the impact index “poor support at work JS5” (compared with the level of impact level 4), and JS5 also had more feedback about the impact of fatigue. Therefore, airlines should increase their support capability for pilots in their work. Figure 1B shows that among the working conditions, the “physical environment JC1 such as noise, temperature, air quality, etc.” and the “transit accommodation/rest conditions JC11” had the most feedback on the impact of fatigue, and JC11 was considered to be the most important factor for fatigue (compared with a 4-level impact). Figure 1C shows that the feedback of the impact index “how much to accomplish what is required to be done W4” in the workload was the highest, and the degree of influence was also the highest. Figure 1D shows that each impact index in the working schedule situation had a high degree of impact on fatigue, and the impact index “ends late in the evening and starts early in the next day A5” had the highest feedback on the impact of fatigue and the highest degree of impact.

### 4.2. Factor analysis

#### 4.2.1. Reliability and validity tests

To determine whether the survey data could be subjected to a factor analysis, it was first necessary to test the reliability and validity of the questionnaire data.

Reliability refers to whether the tools used in the research can stably measure the things or variables being measured; that is, the higher the consistency and stability of the measurement results, the higher the reliability of the research tools used. In this study, the α coefficient was also used to test the reliability of the questionnaire. The α coefficient was between 0 and 1, and the closer it was to 1, the better the reliability of the questionnaire, and the greater the reliability of its measurement results (44). Table 2 shows the reliability test of the questionnaire regarding the factors affecting the fatigue of pilots in terms of the “work” dimension. Table 2 shows that the α coefficient values of the working state factors, working condition factors, workload factors and working schedule factors that affect the fatigue of long-haul flight pilots were 0.917, 0.959, 0.763, and 0.796, respectively, all of which were < 0.7. Therefore, the questionnaire passed the reliability test; that is, the reliability of the data collected in this study was relatively good, and the next step of verification and analysis of influencing factors could be conducted.

**Table 2:** Reliability and validity test of the questionnaire on factors affecting the fatigue of long-haul flight pilots.

**“Work” dimension factor**	**Items**	**α coefficient**	**KMO values**	**χ^2^ of Bartlett's test**
Working status	7	0.917	0.896	0.000
Working conditions	13	0.959	0.958	0.000
Workload	6	0.763	0.789	0.000
Working schedules	6	0.796	0.759	0.000

Validity refers to the degree to which a measurable result conforms to the expected outcome of a psychological or behavioral trait. Construct validity refers to the degree of correspondence between a certain structure reflected in the measurement results and the measurement values. Most studies use factor analysis to extract some common factors that represent the basic structure of the questionnaire. An important indicator of the construct validity test is used to judge whether the questionnaire items are suitable for factor analysis. The test in this aspect usually refers to the KMO value and Bartlett's sphericity test index. The KMO value is between 0 and 1, with 0.5 as the cut-off; values >0.5 and closer to 1 indicate that it is more suitable for a factor analysis. Bartlett's sphericity test is used for judgement analysis based on the judgement standard of Bartlett's test results (that is, the significance probability of its χ^2^ statistic value is < 0.05, indicating that the data are correlated) (45). This study conducted a factor analysis based on the survey data and SPSS analysis software and used the KMO value and the significant probability of the χ^2^ statistical value from Bartlett's sphericity test to test the correlation of the item variables of the long-haul pilot “work” dimension questionnaire and determine whether a factor analysis could be performed. The correlation test results are shown in Table 2.

Table 2 shows that the KMO values of working status, working conditions, workload and working schedules were 0.896, 0.958, 0.789, and 0.759, respectively, all of which were >0.5, indicating that a factor analysis was suitable. The significance probability of the χ^2^ statistical value of Bartlett's test was 0.000, which was far < 0.05, indicating that the data were correlated. It can be seen from the survey results in Table 2 that the data collected in this study were correlated, and the variables could be subjected to a factor analysis. The factor analysis was divided into an exploratory factor analysis (EFA) and confirmatory factor analysis (CFA).

#### 4.2.2. Exploratory factor analysis

An EFA can be used to extract some common factors from all the variables (items) of the scale. If each common factor is highly correlated with a specific question, these common factors represent the basic structure of the scale. Therefore, an EFA can be used to examine whether the designed questionnaire can measure a certain structure assumed at the time of design (46).

The EFA conducted in this study focused on the following two points.

1) The number of common factors to be extracted was determined according to a certain standard. In this study, the cumulative variance contribution rate (that is, the accumulation of the variance percentage, which is the explanation strength of the common factor for the variance of the scale) was set to more than 80% to 85% as the standard (it was considered that the amount of information retained to explain the observed variables was sufficient, and the loss was less, which was a relatively satisfactory result).
2) The interpretability of common factors was considered, and factor rotation was performed to find the best explanation. In this study, the extraction results and component matrices of long-haul pilots' fatigue characteristic common factors were analyzed according to standard criteria with a factor loading value of 0.50 (the percentage of explanatory variables was 20 to 30%; the index variables were close to “good”).

##### 4.2.2.1. Factor analysis of “working status”

Table 3 shows the total variance interpretation table of the data on fatigue conditions affected by working status. It can be seen from the table that when three common factors were extracted, the cumulative variance contribution rate reached 84.265%, in line with the extraction common factor standard set in this study. Therefore, the factor analysis of working status should extract 3 common factors.

**Table 3:** Evaluation parameters when extracting common factors for fatigue influencing factors of the “work” dimension^*^.

**“Work” dimension factor**	**Number of common factors**	**Initial eigenvalues**			**Extract the load sum of squares**			**Rotational load sum of squares**		
		**Total**	**Optional variance percentage**	**Accumulation** **%**	**Total**	**Optional variance percent**	**Accumulation** **%**	**Total**	**Optional variance percent**	**Accumulation** **%**
Working status	1	4.738	67.680	67.680	4.738	67.680	67.680	2.345	33.498	33.498
	2	0.618	8.825	76.505	0.618	8.825	76.505	1.976	28.235	61.734
	3	0.543	7.760	84.265	0.543	7.760	84.265	1.577	22.531	84.265
Working conditions	1	8.872	68.247	68.247	8.872	68.247	68.247	3.659	28.146	28.146
	2	0.681	5.239	73.486	0.681	5.239	73.486	3.155	24.266	52.412
	3	0.610	4.689	78.175	0.610	4.689	78.175	2.374	18.261	70.673
	4	0.464	3.572	81.746	0.464	3.572	81.746	1.439	11.073	81.746
Workload	1	2.827	47.116	47.116	2.827	47.116	47.116	1.555	25.922	25.922
	2	1.014	16.904	64.020	1.014	16.904	64.020	1.344	22.401	48.323
	3	0.680	11.340	75.359	0.680	11.340	75.359	1.176	19.607	67.930
	4	0.573	9.551	84.910	0.573	9.551	84.910	1.019	16.980	84.910
Working schedule	1	2.985	49.742	49.742	2.985	49.742	49.742	1.551	25.853	25.853
	2	0.905	15.086	64.828	0.905	15.086	64.828	1.312	21.859	47.712
	3	0.666	11.101	75.929	0.666	11.101	75.929	1.226	20.436	68.148
	4	0.647	10.775	86.704	0.647	10.775	86.704	1.113	18.556	86.704

^*^Extraction method: principal component analysis.

**Figure 2:**
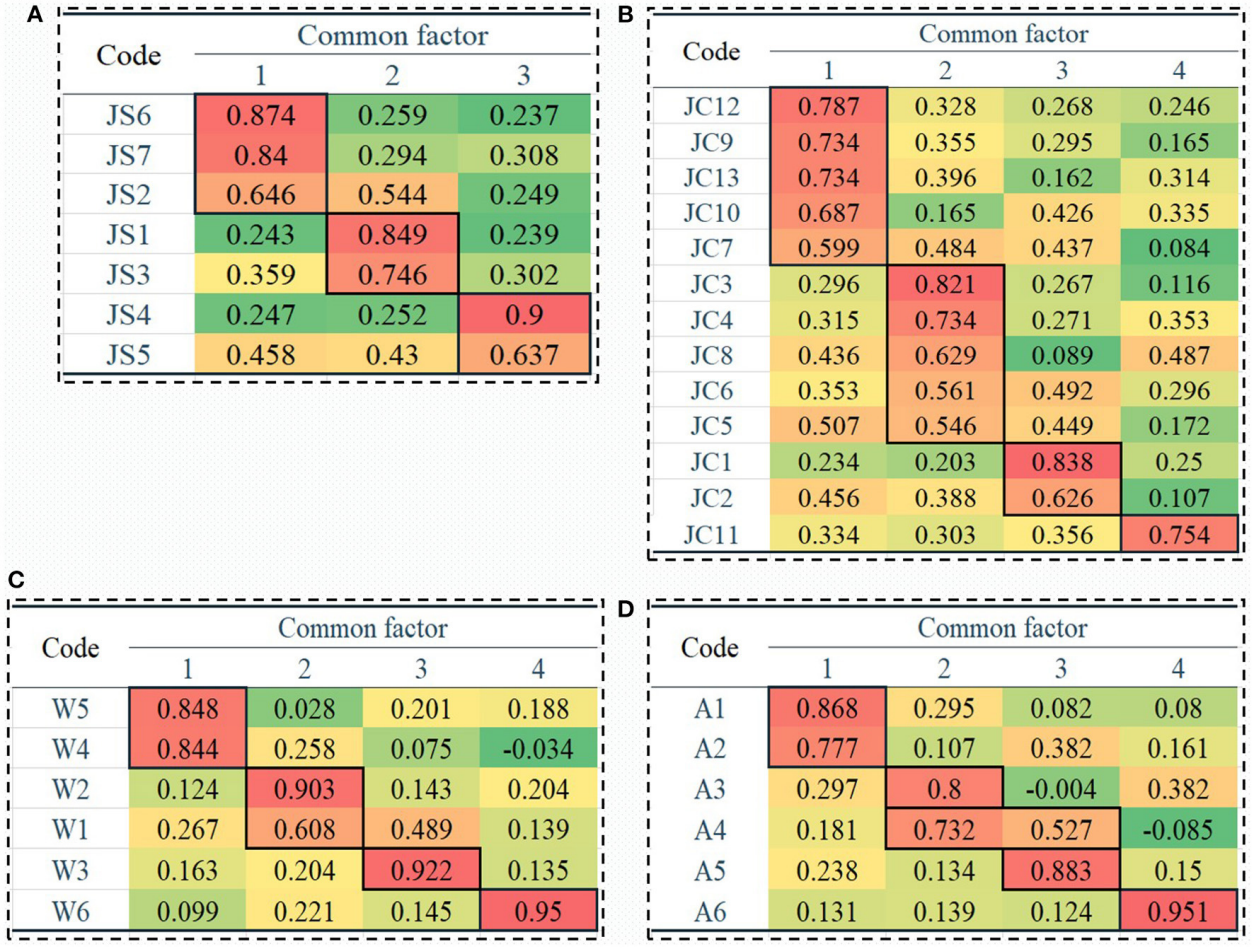
The matrix of common factor load values for each fatigue-influencing factor in the “work” dimension. Extraction method: principal component analysis; Rotation method: Kaiser normal maximum variance method. The common factor matrix after the above rotation: **(B, D)** are the convergent rotations after 5 iterations; **(A, C)** indicate that the rotation has converged after 7 iterations.

Figure 2A shows the rotated load value matrix of 3 common factors for the factor analysis of the affect of working status on fatigue conditions. It can be seen from the figure that the load of “risk brought by passengers JS2” was higher than 0.5 in both dimensions at the same time, indicating it was an invalid item and should be deleted. The load of common factor 1 was mainly concentrated in “conflict within crew JS6” (0.874) and “conflict with ground crew JS7” (0.840), and common factor 1 could be regarded as the “communication/coordination” factor. The load of common factor 2 was mainly concentrated in “interference from the external environment (weather, airflow, etc.) JS1” (0.849) and “risk caused by technology failure, etc., JS3 (0.746)”, and common factor 2 could be regarded as the “emergency situation” factor. The load of common factor 3 was mainly concentrated in “flight plan (temporary) adjustment JS4” (0.900) and “poor support and guarantee at work JS5” (0.637), and common factor 3 could be regarded as the “arrangement guarantee” factor.

##### 4.2.2.2. Factor analysis of “working conditions”

Table 3 shows that when four common factors were extracted from working conditions, the cumulative variance contribution rate reached 81.746%, which met the standard of extraction of the common factors set in this study. Therefore, the factor analysis of working conditions should extract 4 common factors.

Figure 2B shows the rotated load value matrix of 4 common factors for the factor analysis of the affect of working conditions on fatigue conditions. It can be seen from the figure that the load of common factor 1 was mainly concentrated in “commercial operation pressure JC12” (0.787), “flight plan restriction JC9” (0.734), “conditions requiring supervision or control JC13” (0.734), “meal quality JC10” (0.687), and “job repeatability JC7” (0.599). Common factor 1 could be regarded as the “company level” factor. The load of common factor 2 was mainly concentrated in “mental load (monitoring, attention, attention stability, etc.) JC3” (0.821), “work rhythm, time pressure JC4” (0.734), “responsibility pressure JC8” (0.629), “busy airport, etc., JC6” (0.561), and “work rhythm was interrupted JC5” (0.546), and common factor 2 could be regarded as the “personal level” factor. The load of common factor 3 was mainly concentrated in “physical environment such as noise, temperature, air quality JC1” (0.838) and “maintaining a fixed posture while driving (sitting in the cockpit and flying an aircraft) JC2” (0.626), and common factor 3 could be regarded as the “external environment” factor. The load of common factor 4 was mainly concentrated in “transit accommodation and rest conditions JC11” (0.754), and common factor 4 could be regarded as the “rest supplement” factor.

##### 4.2.2.3. Factor analysis of “workload”

Table 3 shows that when four common factors were extracted from workload, the cumulative variance contribution rate reached 84.910%, which met the standard for extracting the common factors set in this study. Therefore, the factor analysis of workload should extract 4 common factors.

Figure 2C shows the rotated load value matrix of the four common factors for the factor analysis of the affect of workload on fatigue conditions. The figure shows that the load of common factor 1 was mainly concentrated in “how hard do you work to reach your current level? W5” (0.848) and “how well do you accomplish what you are asked to do? W4” (0.844), so common factor 1 could be considered the “performance and effort” factor. The load of common factor 2 was mainly concentrated in “how much physical labor does flying demand? W2” (0.903) and “how much mental labor does flying demand? W1” (0.608), so common factor 2 could be regarded as the “energy requirement” factor. The load of common factor 3 was mainly concentrated in “how busy are you after completing each step of the flight? W3” (0.922), and common factor 3 could be regarded as the “time requirement” factor. The load of common factor 4 was mainly concentrated in “how insecure, discouraged, irritable, stressful and troubled are you? W6” (0.950), and common factor 4 could be regarded as the “frustration feeling” factor.

##### 4.2.2.4. Factor analysis of “working schedule”

It can be seen from Table 3 that when four common factors were extracted, the cumulative variance contribution rate reached 86.704%, which met the standard of extraction of the common factors set in this study. Therefore, four common factors should be extracted from the factor analysis of the impact of shift schedule on fatigue.

Figure 2D shows the rotated load value matrix of the four common factors for the factor analysis of the affect of working schedule on fatigue conditions. It can be seen from the figure that the load of “night flight with a transit time longer than 6 h A4” was higher than 0.5 in both dimensions at the same time, so it was an invalid item and should be deleted. The load of common factor 1 was mainly concentrated in “international route (exemption, no overnight) A1” (0.868) and “segment mission with transit time between 2 and 4 h A2” (0.777), and common factor 1 could be regarded as the “no overnight exemption” factor. The load of common factor 2 was mainly concentrated in “on duty time exceeding 10 h A3” (0.800), and common factor 2 could be regarded as the “long duty” factor. The load of common factor 3 was mainly concentrated in “the end of the evening is late, and the next day starts early by A5” (0.883), and common factor 3 could be regarded as the “insufficient rest” factor. The load of common factor 4 was mainly concentrated in “international routes (not exempt, overnight) A6” (0.951), and common factor 4 could be regarded as the “exempt from overnight stay” factor.

#### 4.2.3. Confirmatory factor analysis

CFA is a statistical analysis of social survey data that tests whether the mutual influence among many factors and their corresponding observations conform to the theoretical relationship designed by the researcher. It is possible to make assumptions about the mutual influence among latent variables and observed variables according to a specific human fatigue theory and then verify the rationality of this assumption. Therefore, CFA is a powerful tool for the construction and verification of theoretical psychological models, such as fatigue, overcoming the shortcomings of EFA. It is also a prestep for conceptual model construction.

**Figure 3:**
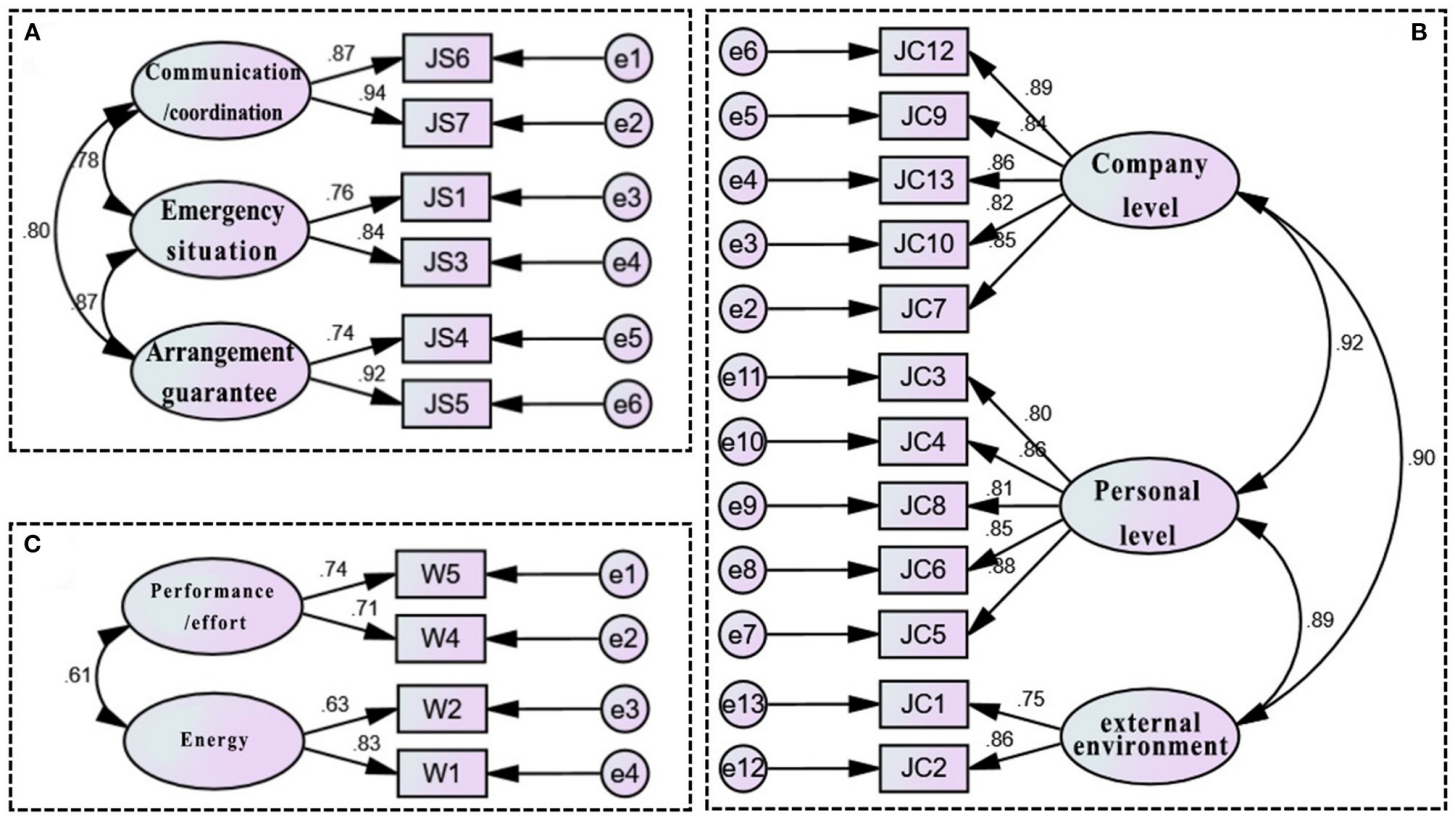
CFA analysis of fatigue-influencing factors in long-haul flight pilots' work dimension. **(A)** Factor of working status, **(B)** factor of working conditions, and **(C)** factor of workload.

**Figure 4:**
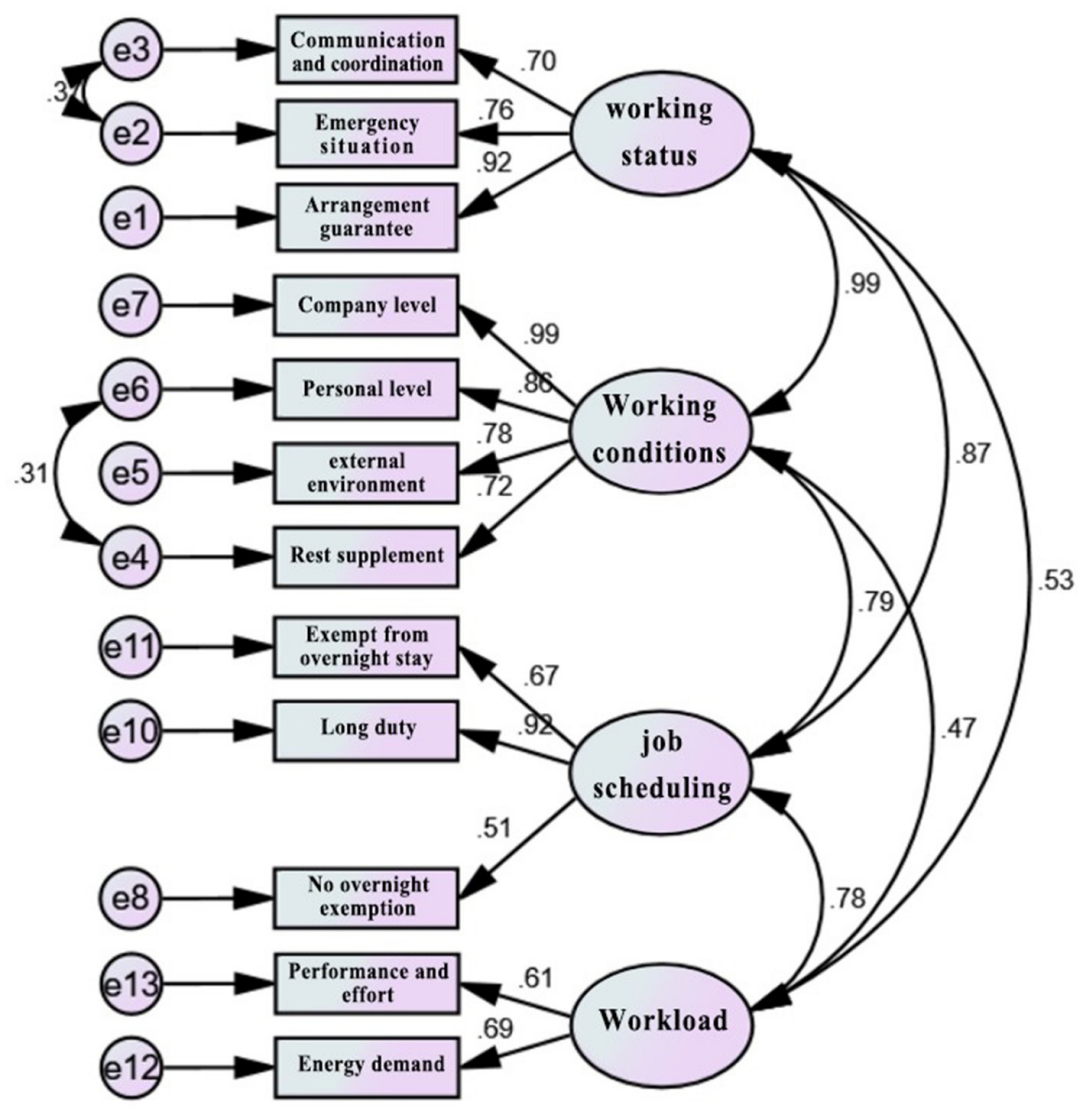
Path analysis model of fatigue-influencing factors of long-haul flight pilots' “work” dimension.

CFA uses a structural equation model (SEM), and its function is to verify the degree of fit between the hypothetical model and the sample data, that is, to evaluate whether the hypothetical model structure is suitable for the sample data. SEM is a research methodology based on statistical analysis technology that can be used to address the exploration and analysis of complex multivariable research data. More importantly, SEM can simultaneously estimate the latent variables and the parameters of the complex independent variable/dependent variable prediction model (47). SEM is also a methodology that uses a validation (i.e., hypothesis testing) approach to the analysis of theories related to certain phenomena, and one of its unique features is a validating approach to data analysis by specifying a priori the relationships between variables (48). In addition, after the model is fitted, the model is validated according to the fit indices (GFI = goodness-of-fit; AGFI =adjusted goodness-of-fit; SRMR=standardized root mean residual; RMAES = root mean square error of approximation; CFI =comparative fit index; TLI = Tucker–Lewis index, etc.), and the best model is determined by continuously correcting the model fit indices to meet the standards (49). SEM using AMOS software was used to construct and analyze the data. Table 4 shows the analysis results of the SEM parameters, and Figures 3, 4 show the relevant SEM verification results.

**Table 4:** CFA of long-haul flight pilots' fatigue characteristics.

**“Work” dimension factor**	**χ^2^/df**	**GFI**	**AGFI**	**NFI**	**RMSEA**
Working status	1.477	0.989	0.963	0.991	0.043
Working conditions	3.538	0.896	0.853	0.938	0.098
Workload	1.462	0.997	0.972	0.994	0.042
Ideal standard value	< 5, < 3 is best	>0.9 or 0.85	>0.9 or 0.85	>0.9 or 0.85	< 0.1, < 0.05 is best

##### 4.2.3.1. Verification of the structural equation measurement model of each fatigue influencing factor in the “work” dimension

Table 4 shows that the value range of the overall fitness index of each structural equation measurement model of working status, working conditions and workload was within the ideal standard value range.

As stated above, the α coefficient is taken as the reliability coefficient of the questionnaire, and the combined reliability in the SEM is taken as the reliability coefficient of the latent variable, which can be used as one of the judgement criteria for measuring the intrinsic quality of the model; that is, when the combined reliability value of the latent variable is >0.6, it can be considered that the intrinsic quality of the measurement model is good.

As seen from Table 5, the combination reliability of all potential variables of the fatigue characteristics of long-haul flight pilots, including working status, working conditions and workload, was >0.6, indicating that, on the whole, the measurement model of fatigue-influencing factors in the “work” dimension of long-haul flight pilots had good intrinsic quality. In addition, Table 5 shows that the AVE (convergent validity) of all of the latent variables of the fatigue characteristics of long-haul flight pilots was >0.5, and the convergent validity was also good.

**Table 5:** Factor loading value, convergent validity and combined reliability of the long-haul flight pilots' fatigue characteristics measurement model.

**“Work” dimension factor**	**Path**			**Estimate**	**S.E**.	**C.R**.	***P***	**AVE**	**CR**
Working status	JS6	< −−−	Communication/coordination	0.87				0.8202	0.9011
	JS7	< −−−	Communication/coordination	0.94	0.058	18.753	^***^		
	JS1	< −−−	Emergency situation	0.755				0.6378	0.7784
	JS3	< −−−	Emergency situation	0.84	0.106	12.678	^***^		
	JS4	< −−−	Arrangement guarantee	0.743				0.6973	0.8201
	JS5	< −−−	Arrangement guarantee	0.918	0.094	13.72	^***^		
Working conditions	JC7	< −−−	Company level	0.851				0.7255	0.9296
	JC10	< −−−	Company level	0.818	0.065	16.725	^***^		
	JC13	< −−−	Company level	0.857	0.076	18.125	^***^		
	JC9	< −−−	Company level	0.839	0.073	17.476	^***^		
	JC12	< −−−	Company level	0.892	0.065	19.519	^***^		
	JC5	< −−−	Personal level	0.875				0.7063	0.9231
	JC6	< −−−	Personal level	0.851	0.057	18.725	^***^		
	JC8	< −−−	Personal level	0.813	0.058	17.227	^***^		
	JC4	< −−−	Personal level	0.857	0.056	19.003	^***^		
	JC3	< −−−	Personal level	0.804	0.061	16.895	^***^		
	JC2	< −−−	External environment	0.857				0.6447	0.7832
	JC1	< −−−	External environment	0.745	0.064	13.44	^***^		
Workload	W5	< −−−	Performance and effort	0.738				0.5265	0.6898
	W4	< −−−	Performance and effort	0.713	0.18	6.371	^***^		
	W2	< −−−	Energy requirement	0.633				0.5456	0.7022
	W1	< −−−	Energy requirement	0.831	0.189	5.91	^***^		

The ^***^ symbol indicates the statistical significance.

Table 6 shows that the correlation coefficient of each common factor of working status, working conditions and workload was less than the square root of the corresponding AVE, proving discriminant validity.

**Table 6:** Statistics of the correlation coefficients between the latent variables of fatigue factors in terms of the “work” dimension of long-haul flight pilots.

	**Communication/coordination**	**Emergency situation**	**Arrangement guarantee**
Communication/coordination	0.8202		
Emergency situation	0.469	0.6378	
Arrangement guarantee	0.61	0.522	0.6973
Square root of AVE	0.905648939	0.798623816	0.835044909
	**Company level**	**Personal level**	**External environment**
Company level	0.7255		
Personal level	0.559	0.7063	
External environment	0.54	0.683	0.6447
Square root of AVE	0.851762878	0.840416563	0.802932127
	**Performance and effort**	**Energy requirement**	
Performance and effort	0.5265		
Energy requirement	0.611	0.5456	
Square root of AVE	0.725603197	0.738647413	

According to the above verification analysis of the structural equation measurement model of each influencing factor of the “work” dimension, each path was verified, and the relevant graphic output of AMOS is shown in Figure 3, that is, the CFA model of each influencing factor of the “work” dimension. Figures 3A–C show the CFA among the indicators of long-haul flight pilots' working status, working conditions and workload, respectively. While the indicators of long-haul flight pilots' working schedule had only one common factor, and there were two factors, the others were all factors, so no CFA was needed.

##### 4.2.3.2. Validation of the SEM of various fatigue-influencing factors in the “work” dimension

According to Table 7, the first calculation in the SEM of the “work” dimension showed that χ^2^/df =5.689 > 5 and AGFI = 0.762 < 0.85, RMSEA = 0.134 >0.1. Therefore, the fitting effect was not ideal, and the model was modified. From the correction index item of the model output result, it was found that when the relevant line was drawn between “e2” and “e3”, the corresponding chi-square value decreased the most (to 32.107), and a first correction was performed. After the correction, it was found that χ^2^/df=3.676, AGFI = 0.817, and RMSEA = 0.101; the fitting effect was still not ideal, and a second correction was performed. When the relevant line was drawn between “e9” and “e11”, the corresponding chi-square value decreased the most (to 20.298). However, the second correction did not change the results much, and another correction method was used. From the model fitting results, it was found that the factor loading factor of insufficient rest in the working schedule was 0.405 < 0.5. This item was deleted for correction. The fitness indices of the fatigue-influencing factors of the “work” dimension of flight pilots all reached the ideal range. Therefore, based on the above analysis, this study concluded that after three revisions of the preliminary SEM of fatigue-influencing factors in the “work” dimension of long-haul flight pilots, the fitting effect of the model was better, so it could be used as the final model for further analysis and interpretation.

**Table 7:** Path analysis of various influencing factors of fatigue in the “work” dimension of long-haul flight pilots and the statistics of each adaptation index of the revised results.

	**χ^2^/df**	**GFI**	**AGFI**	**NFI**	**RMSEA**	**Possible reasons why the model is not ideal**
Before correction	4.512	0.858	0.782	0.894	0.116	e2–e3/32.107
First correction	3.986	0.873	0.801	0.908	0.107	e9–e11/20.298
Second correction	3.676	0.885	0.817	0.917	0.101	Lack of rest – Working schedule/0.405; e4–e6/20.088
Third amendment	3.447	0.908	0.844	0.935	0.096	Well

As seen in Table 8, after three corrections, the combination reliability of fatigue-influencing factors in the “work” dimension of long-haul flight pilots was >0.6, indicating that, on the whole, the SEM of fatigue-influencing factors in the “work” dimension of long-haul flight pilots had good intrinsic quality. The AVE (convergent validity) of fatigue-influencing factors in the “work” dimension was >0.5, and the convergent validity was also good.

**Table 8:** Factor loading value, convergent validity and combined reliability information of the path analysis model of fatigue-influencing factors in the “work” dimension of long-haul flight pilots.

**Path**			**Estimate**	**SE**	**CR**	***P***	**AVE**	**CR**
Arrangement guarantee	< −−−	Working status	0.924				0.637	0.8384
Emergency situation	< −−−	Working status	0.755	0.056	16.759	^***^		
Communication/coordination	< −−−	Working status	0.698	0.049	14.582	^***^		
Rest supplement	< −−−	Working conditions	0.72				0.711	0.9065
External environment	< −−−	Working conditions	0.776	0.123	12.77	^***^		
Personal level	< −−−	Working conditions	0.861	0.271	16.698	^***^		
Company level	< −−−	Working conditions	0.991	0.288	16.311	^***^		
No overnight exemption	< −−−	Working schedule	0.511				0.5198	0.7545
Long duty	< −−−	Working schedule	0.918	0.194	8.684	^***^		
Exempt from overnight stay	< −−−	Working schedule	0.675	0.253	7.669	^***^		
Energy requirement	< −−−	Workload	0.693				0.425	0.5954
Performance and effort	< −−−	Workload	0.608	0.126	7.169	^***^		

The ^***^ symbol indicates the statistical significance.

It can be seen from Table 9 that after three corrections, the mutual coefficients between the factors affecting the fatigue of long-haul flight pilots' “work” dimension were smaller than the square root of the corresponding AVE, so the model had good discriminant validity.

**Table 9:** Correlation coefficients between factors affecting fatigue in long-haul flight pilots' “work” dimension.

	**Working status**	**Working conditions**	**Working schedule**	**Workload**
Working status	0.637			
Working conditions	0.992	0.711		
Working schedule	0.873	0.789	0.5198	
Workload	0.528	0.466	0.785	0.425
Square root of AVE	0.798122798	0.843208159	0.720971567	0.651920241

Established through the long-haul flight pilot “work” dimension SEM validation of the factors affecting fatigue and on the basis of the fitting test evaluation to constantly revise and improve the model, the model fitting effect was rendered perfect, and through final inspection, the final form of the SEM was provided, as shown in Figure 4.

##### 4.2.3.3. The mutual influence among the fatigue influencing factors of the “work” dimension and their respective indicators

In terms of working status, according to Figure 4, the load of the “arrangement guarantee” factor in working status was the largest (0.92), followed by the “emergency situation” factor (0.76) and the “communication/coordination” factor (0.70). Therefore, combined with the analysis of working status in Section 4.2.2 *via* EFA, it can be seen that compared with the “communication/coordination” factor “conflict within crew JS6” and “conflict with ground crew JS7”and the “emergency situation” factor, such as “interference from the external environment JS1”/ “risk caused by technology failure JS3”, company-level arrangement support factors, such as “flight plan adjustment JS4” and “poor support and guarantee at work JS5” had a greater impact on pilot fatigue.

In terms of working conditions, it can be seen from Figure 4 that the “company level” had the largest loading (0.99), followed by the “personal level” factor (0.86), the “external environment” factor (0.78) and finally the “rest supplement” factor (0.72). Combined with the analysis of working conditions in Section 4.2.2's EFA, it can be seen that the “company level” factor's common factors extracted from JC7, JC9, JC13, JC12, and JC10 greatly impacted pilot fatigue. Therefore, providing better working conditions at the company level could reduce the risk of fatigue for pilots on long-haul flights.

In terms of workload, it can be seen from Figure 4 that the loads of the workload factors were all < 0.7, and the largest factor was the “energy requirement” factor (0.69). Combined with the workload analysis in Section 4.2.2's EFA, it can be seen that, compared with the busy work (W3) and workload feelings (W6, W4, and W5) indices, the “energy requirement” common factors physical requirement (W2) and mental requirement (W1) indices had relatively greater impacts on pilot fatigue.

In terms of working schedule, Figure 4 shows that the load of the “long duty” factor for working schedule was the largest (0.92), followed by whether the destination is overnight or not, while the “no overnight exemption” and “exempt from overnight stay” factors were smaller (0.67 and 0.51, respectively). Combined with the working schedule analysis in Section 4.2.2's EFA, it can be seen that “longer duty with more than 10 h of duty time A3” had a greater impact on the fatigue of long-haul flight pilots.

##### 4.2.3.4. The mutual influence among fatigue-influencing factors in the “work” dimension

Figure 4 shows that among the mutual influences of the factors in the “work” dimension, the correlation between working status and working conditions was the largest (0.99), the correlation between working status and working schedule was also relatively large (0.87), and the correlation between working status and workload was small (0.53); that is, the long-haul flight pilots' working status, working schedules and working conditions had strong mutual influences, and the influence between workload and working status was small. Therefore, Hypotheses a and b were established, and Hypothesis c was not established. The correlation between working conditions and working schedule was large (0.79), and the correlation between working conditions and workload was small (0.47); that is, there was a strong mutual influence between long-haul flight pilots' working conditions and working schedules, and the mutual influence between working conditions and workload was small; therefore, Hypothesis d was established, and Hypothesis e was not established. The correlation between working schedules and workload was also large (0.78); that is, the mutual influence between the workload and working schedule of long-haul flight pilots was large, and thus, Hypothesis f was established.

## 5. Conclusions

This study is the first to focus on one of the causes of pilot fatigue—the influencing factors of the “work” dimension. Based on the analysis and summary of the relevant literature, we proposed hypotheses about the mutual influence among various fatigue-influencing factors in the pilot “work” dimension, and a new questionnaire was developed to assess the mutual influence among factors influencing fatigue in the pilot “work” dimension. Then, surveys were conducted among groups of long-haul flight pilots to obtain real empirical data on actual flight operation scenarios.

In terms of questionnaires, the basic information of pilots was first included for descriptive statistical analysis, limiting the relevant results of this study to focus on explaining the characteristics of copilots, married pilots, those with one child, exempted flights, and long-haul flights to the Americas. Then, a total of 32 items were divided into 4 subscales: working status, working conditions, workload and working schedule. Finally, reliability and validity tests of the questionnaire, as well as an EFA and CFA, were performed. The results showed that the reliability of the questionnaire was better tested by the alpha coefficient of the pilot survey data. The KMO value and Bartlett's sphericity test were used to test the correlation of the questionnaire item variables, and it was verified that the questionnaire had good validity and that the survey data were correlated and thus were suitable for a factor analysis.

In terms of the EFA, first, basic pilot information was included for descriptive statistical analysis, in line with other questionnaires, with basic information about the subjects (50). Then, 32 questions were included in four dimensions: work status (51–53), workload (50), work schedule (54, 55) and working conditions (56, 57). Finally, the questionnaire was subjected to reliability and validity tests (58, 59), as well as an EFA and validation factor analysis (60, 61). The results showed that the alpha coefficient of the pilot survey data tested the reliability of the questionnaire, and the KMO value and Bartlett's spherical test were used to test the correlation of the questionnaire's question variables, which showed that the questionnaire had good validity and that the survey data were correlated and suitable for a factor analysis. In addition, the results of the mutual influence among the fatigue factors of the long-haul flight pilots' “work” dimension showed that for long-haul flight pilots, their working status, working conditions, and working schedules had a strong mutual influence, working schedule and workload also had a strong mutual influence, and the mutual influence between working status and workload, working conditions and workload was weak.

Although this study conducted a detailed analysis of the mutual influence of various factors affecting the fatigue of pilots' “work” dimension, the results are expected to help regulators or airline safety management departments increase aviation safety, such as helping them to develop aviation-related safety regulations or guidelines (11, 25, 26). However, similar to other research efforts, this study has limitations. For example, the results of this study are biased toward empirical data obtained from co-pilot respondents on long-haul flights, so the results might not be sufficiently generalizable to a larger group of pilots in the captain category. And this study did not consider the distinction between the types of work across time zones and north–south across longitudes in long-haul flights, and it is hoped that a distinction can be made in future research.

## Data availability statement

The original contributions presented in the study are included in the article/Supplementary material, further inquiries can be directed to the corresponding authors.

## Ethics statement

The studies involving human participants were reviewed and approved by the Institutional Review Board (IRB) at Civil Aviation University of China. The patients/participants provided their written informed consent to participate in this study.

## Author contributions

SJ-Y conceived and designed the experiments, performed the experiments, analyzed the data, prepared the figures and/or tables, authored or reviewed drafts of the paper, and approved the final draft. SR-S conceived and designed the experiments, analyzed the data, prepared the figures and/or tables, authored or reviewed drafts of the paper, and approved the final draft. All authors contributed to the article and approved the submitted version.
