# Supplementary material for: Pilot fatigue survey: A study of the mutual influence among fatigue factors in the “work” dimension
Source: Front Public Health. 2023 Feb 2;11:1014503. doi: 10.3389/fpubh.2023.1014503 (PMC9932798; doi:10.3389/fpubh.2023.1014503)
Supplement: Supplementary file 1 [file Table_1.DOCX]

Appendix

1. Basic personal information (please fill in "_" as required, or tick "√" in "□" after the option)

sex：_____ age：_____

who are you? □ instructor  □ captain □ co-pilot

marital status： □ married □ unmarried □ divorced □ widowed

The number of children： □none □one □ Two or more

Hours of employment with this (subsidiary) company： ______ (e.g:105 months)

Total flight hours： _____ hours

Number of flight hours for this type of aircraft： _____ hours

commuting time： _____min

Types of routes operated/serviced (major)： □ International routes (exemption/no overnight) □ International routes (overnight) □ Domestic Routes (High Plateau Airport)

□ Domestic Routes (other routes)

The flight path area of this flight： 🞎 America 🞎 Europe 🞎 Australia

1. In your opinion, do the following working status affect fatigue and to what extent? (The second column can be selected multiple times. After selecting this factor, evaluate the impact of this factor on fatigue, and tick "√" in the corresponding space)

| Coding | Items | slight influence  （1） | a little influence  （2） | Moderate influence  （3） | significant influence  （4） | deep  influence（5） |
| --- | --- | --- | --- | --- | --- | --- |
| JS1 | □ Weather, air flow and other environmental disturbances |  |  |  |  |  |
| JS2 | □ Risks posed by passengers |  |  |  |  |  |
| JS3 | □ By technology, failure and other risks |  |  |  |  |  |
| JS4 | □ A (temporary) adjustment of the flight schedule |  |  |  |  |  |
| JS5 | □ Poor support at work |  |  |  |  |  |
| JS6 | □ Internal conflict among the crew |  |  |  |  |  |
| JS7 | □ Problems with people on the ground |  |  |  |  |  |

1. In your opinion, do the following working conditions affect fatigue and to what extent? (The second column can be selected multiple times. After selecting this factor, evaluate the impact of this factor on fatigue, and tick "√" in the corresponding space)

| Coding | Items | slight influence（1） | a little influence（2） | Moderate influence  （3） | significant influence  （4） | deep influence（5） |
| --- | --- | --- | --- | --- | --- | --- |
| JC1 | □ Noise, temperature, air quality, etc |  |  |  |  |  |
| JC2 | □ Keep a fixed position while driving |  |  |  |  |  |
| JC3 | □ Mental load (monitoring, attention, stability of attention, etc.) |  |  |  |  |  |
| JC4 | □ Work pace, time pressure |  |  |  |  |  |
| JC5 | □ The work rhythm is interrupted |  |  |  |  |  |
| JC6 | □ Busy airport, etc. |  |  |  |  |  |
| JC7 | □ Repeatability of work |  |  |  |  |  |
| JC8 | □ social duty and pressure |  |  |  |  |  |
| JC9 | □ Constraints on flight schedules |  |  |  |  |  |
| JC10 | □ Eating quality |  |  |  |  |  |
| JC11 | □ Over stop accommodation/rest conditions |  |  |  |  |  |
| JC12 | □ Commercial operational pressure |  |  |  |  |  |
| JC13 | □ A condition requiring supervision or control |  |  |  |  |  |

1. In your opinion, do the following workload affect fatigue and to what extent? (The second column can be selected multiple times. After selecting this factor, evaluate the impact of this factor on fatigue, and tick "√" in the corresponding space)

| Coding | Items | slight influence  （1） | a little influence  （2） | Moderate influence  （3） | significant influence  （4） | deep influence  （5） |
| --- | --- | --- | --- | --- | --- | --- |
| W1 | □ How mentally demanding is this flight? |  |  |  |  |  |
| W2 | □ How physically demanding was the flight? |  |  |  |  |  |
| W3 | □ How rushed were you to complete each step of the flight? |  |  |  |  |  |
| W4 | □ To what extent have you done what you've been asked to do? |  |  |  |  |  |
| W5 | □ How hard have you worked to get to where you are? |  |  |  |  |  |
| W6 | □ How insecure, discouraged, irritable, stressed and annoyed are you? |  |  |  |  |  |

1. In your opinion, do the following working schedules affect fatigue and to what extent? (The second column can be selected multiple times. After selecting this factor, evaluate the impact of this factor on fatigue, and tick "√" in the corresponding space)

| Coding | Items | slight influence  （1） | a little influence  （2） | Moderate influence  （3） | significant influence  （4） | deep  influence（5） |
| --- | --- | --- | --- | --- | --- | --- |
| A1 | □International routes (exemption/no overnight) |  |  |  |  |  |
| A2 | □Segment tasks with an overpass time between 2 and 4 hours |  |  |  |  |  |
| A3 | □More than 10 hours on duty |  |  |  |  |  |
| A4 | □A night flight with an overpass time of more than 6 hours |  |  |  |  |  |
| A5 | □The night ends late and the day begins early. |  |  |  |  |  |
| A6 | □International routes (not exempt, overnight) |  |  |  |  |  |
